# Alterations in Peripheral and Central Components of the Auditory Brainstem Response: A Neural Assay of Tinnitus

**DOI:** 10.1371/journal.pone.0117228

**Published:** 2015-02-19

**Authors:** Andrea S. Lowe, Joseph P. Walton

**Affiliations:** 1 Department of Communication Sciences & Disorders, University of South Florida, Tampa, Florida, United States of America; 2 Department of Chemical & Biomedical Engineering, University of South Florida, Tampa, Florida, United States of America; 3 Global Center for Hearing & Speech Research, University of South Florida, Tampa, Florida, United States of America; University of Texas at Dallas, UNITED STATES

## Abstract

Chronic tinnitus, or “ringing of the ears”, affects upwards of 15% of the adult population. Identifying a cost-effective and objective measure of tinnitus is needed due to legal concerns and disability issues, as well as for facilitating the effort to assess neural biomarkers. We developed a modified gap-in-noise (GIN) paradigm to assess tinnitus in mice using the auditory brainstem response (ABR). We then compared the commonly used acoustic startle reflex gap-prepulse inhibition (gap-PPI) and the ABR GIN paradigm in young adult CBA/CaJ mice before and after administrating sodium salicylate (SS), which is known to reliably induce a 16 kHz tinnitus percept in rodents. Post-SS, gap-PPI was significantly reduced at 12 and 16 kHz, consistent with previous studies demonstrating a tinnitus-induced gap-PPI reduction in this frequency range. ABR audiograms indicated thresholds were significantly elevated post-SS, also consistent with previous studies. There was a significant increase in the peak 2 (P2) to peak 1 (P1) and peak 4 (P4) to P1 amplitude ratios in the mid-frequency range, along with decreased latency of P4 at higher intensities. For the ABR GIN, peak amplitudes of the response to the second noise burst were calculated as a percentage of the first noise burst response amplitudes to quantify neural gap processing. A significant decrease in this ratio (i.e. recovery) was seen only at 16 kHz for P1, indicating the presence of tinnitus near this frequency. Thus, this study demonstrates that GIN ABRs can be used as an efficient, non-invasive, and objective method of identifying the approximate pitch and presence of tinnitus in a mouse model. This technique has the potential for application in human subjects and also indicates significant, albeit different, deficits in temporal processing in peripheral and brainstem circuits following drug induced tinnitus.

## Introduction

Chronic tinnitus, an auditory perception not attributable to an external source, affects between 4% and 15% of adults and increases with age [1]. It is also the most-reported service-related disability for veterans returning from Middle Eastern conflicts, with almost 1 million veterans receiving military compensation annually for tinnitus [2]. Because self-reporting of tinnitus is by nature subjective, an objective and comprehensive measure is highly desirable for diagnostic, legal, and disability determination [3]. Recent advances in brain imaging techniques have proven useful in identifying neural correlates of tinnitus, but they have not yet proven reliable, cost-effective, or efficient for clinical assessments [3]. Here, we present evidence for the potential of an objective method for tinnitus evaluation using the auditory brainstem response (ABR).

Several studies using animal models have reported variations in parameters of the ABR, such as peak amplitudes or responses to maskers, after employing methods that induce tinnitus [4–6]. None of these have proven suitable as a replacement for current behavioral test methods, however. Behavioral assays include Pavlovian lick training [7], assessing noise-rewarded feeder access [8], and schedule-induced polydipsia avoidance conditioning [9]. The shortcoming of these methods is that they involve fairly time-consuming training. This limitation is not shared by measuring gap detection deficits using pre-pulse inhibition (PPI) of the acoustic startle response (ASR) in a gap-in-noise (GIN) paradigm to determine if tinnitus is present [10–14]. In the GIN paradigm, the insertion of a silent gap in a frequency centered carrier noise before a startle eliciting signal (SES) inhibits the ASR [15]. The efficacy of a gap to inhibit the ASR reflects the animal’s ability to detect the gap. When the gap carrier is qualitatively similar to the animal’s tinnitus pitch, the gap-PPI of the ASR is reduced, as the tinnitus hypothetically “fills in” the gap [11]. Several research groups have adopted the use of the gap-startle paradigm and proposed that it is efficient tool to diagnosis tinnitus, since it does not require animal training and allows for approximation of the tinnitus pitch. While the GIN approach has advantages, there are also limitations, including vulnerability to changes in animal stress levels, difficulty inducing ASRs in older or hearing-impaired subjects, and the necessity of robust ASRs over multiple test sessions for an accurate response calculation [16]. It has also been reported that transient, unilateral conductive hearing loss also resulted in impaired gap-PPI, indicating the risk of a false positive assessment of tinnitus [17]. The use of ABR recordings can overcome these limitations as well as those faced by conditioning paradigms.

To assess methods for identifying the presence and pitch of tinnitus in animal models, tinnitus must first be reliably induced. Administering acute doses of sodium salicylate (SS), the active ingredient in aspirin, is widely used as a consistent method of tinnitus induction in animal models [7,18–20]. Testing 1–2 hours following SS injection in rats has been shown to impair GIN detection when the gap carrier is in the 16 kHz range [21,22], suggesting that the induced tinnitus percept is in the 16 kHz region. While there is a paucity of data regarding ABR tone intensity function shifts or threshold changes immediately following SS-induced hearing loss and tinnitus, considerable research has been performed examining post-SS threshold shifts within specific auditory structures. An earlier study determining threshold changes based on the ABR peak 1 (P1) amplitude found an increase of approximately 12–20 dB SPL (dependent of stimulus frequency) following aspirin administration in rats [23]. When the compound action potential (CAP), an assessment of cochlear sensitivity, was measured in rats before and after systemic SS treatment, a significant increase in the thresholds at all tested frequencies was observed along with a significant reduction in CAP response amplitude, although the amplitude reduction was least at 16 kHz [24].

The ABR is an acoustically stimulated electrophysiological signal that represents activity from the cochlea, auditory nerve (AN), and brainstem, and is measured non-invasively. The peripheral evoked potentials from the cochlear hair cells and AN are believed to contribute to P1 of the ABR in rodents, with the cochlear nucleus (CN) contributing to peak 2 (P2) [25]. Peak 4 (P4) in rodent models is thought to parallel wave V of the human ABR [26], a generally robust wave assumed to originate in the vicinity of the lateral lemniscus/inferior colliculus (IC) [27]. ABR responses to GIN paradigms were examined by Boettcher et al., who found that as the silent gap between two low-pass noises increased, ABR peak amplitudes to the second stimulus increased and there was also almost complete recovery of the second response when the gap duration reached 32 ms. Boettcher et al. attributed the peak amplitude recovery from prior stimulation to recovery observed at the level of the auditory nerve which is specifically related to synaptic transmission at the inner hair cell-nerve synapse. They also found evidence that this recovery ratio was not affected by age-related hearing loss in gerbils [28]. This is important in tinnitus studies of GIN, as Nouvian et al. states that the inner hair cell (IHC) presynaptic active zone may contribute to tinnitus induction, due to increases in excitability in the auditory fiber firing rate [29]. In mice, sensorineural hearing loss increased the time constant of ABR peak 5 (P5) amplitude forward masking recovery functions and increased peak latency as compared to normal hearing mice [30].

The goal of the present study was to determine if changes in ABR response metrics could be used as an objective and reliable method for assessing tinnitus in animal models. The basic gap-PPI circuit has been attributed to neural activity in the brainstem [31], therefore it is highly probable that the ABR could be used to assay tinnitus induced changes. To our knowledge, we are the first to report the use of the GIN ABR paradigm in the assessment of tinnitus in an animal model. We first confirmed previous behavioral findings of frequency specific gap-PPI reductions, as well as a trend towards increased ASR amplitudes following SS treatment. We then measured the amplitudes and latencies for P1, P2, and P4 of ABR responses to the tone intensity function and GIN signals. Recovery following a gap in the ABR response amplitude for these peaks was also examined. These ABR peaks were chosen for analysis because they are easily identified in the mouse ABR, and can give measures of post-SS changes in both the peripheral and central auditory system, indicating if central amplification is occurring. Latency was also assessed in both the ASR and ABR to determine if tinnitus altered neurotransmission through the brainstem.

## Methods

### Animals

Subjects were 12 young adult (2–4 months old) CBA/CaJ mice (4 males, 7 females), used longitudinally as both control and treated animals. Initial testing did not include tone intensity functions or certain frequencies of the GIN paradigm; therefore some tests are reported with n = 8. Mice were obtained from the Jackson Laboratory (Bar Harbor, ME) at 6 weeks of age and housed 3–4 per cage with litter-mates in Sealsafe Plus GM500 cages (36 x 16 x 13 cm) connected to a Aero70 Techniplast Smart Flow system (West Chester, PA). The housing cages were maintained at a constant temperature and humidity, using a 12 hour light cycle with water and food pellets available *ad libitum*. All procedures were approved by the Institutional Animal Care and Use Committee at the University of South Florida (IACUC #M3847).

### Behavioral testing

Prior to testing, each animal’s home cage was placed in the testing room for 30 min to allow for acclimation to the surroundings, with assessment of only one sex occurring in a single day. Mice were individually tested in a wire mesh cage (9.5 x 4 x 4 cm), resting on a custom built platform connected to piezoelectric transducers, which was located inside one of four identical sound attenuated chambers (40 x 40 x 40 cm). Animals were given 5 min for acclimation before testing. Each animal received 3–6 testing sessions over the course of one week. If a mouse was tested more than once in a single day, a rest period in their home cage was provided for at least 1 hour between sessions.

SESs were filtered (500 Hz—40 kHz), 20 ms, 115 dB SPL Gaussian broadband noise bursts (1 ms rise/fall time) presented at pseudorandom inter-trial intervals between 10 and 20 seconds. For gap-PPI testing, the SES was preceded by a 150 ms 70 dB narrow band noise (1/3 octave) centered at 6, 12, 16, 20, or 24 kHz presented with or without a 50 ms silent gap inserted 100 ms before the SES. For each noise band, 20 trials were presented with a gap and 20 trials without a gap in a pseudo-random order. Comparison of ASR amplitude with and without the gap provided a measure of gap detection. Acoustic stimuli were presented through Fostex model FT17H speakers (Fostex Company, Tokyo, Japan) located 30 cm directly above the transducer platform and controlled with a RZ6 multi-I/O processor from Tucker-Davis Technologies (TDT, Alachua, FL) and custom MATLAB software (The MathWorks, Inc., Matick, MA). All signals were calibrated prior to testing with a 1/4” microphone placed at the level of the animal’s pinna in the ASR chamber and led to a Larsen Davis preamplifier, model 2221 (PCB Piezotronics, Inc., Depew, NY). Transducer responses to movement (in millivolts) were recorded over the time period 125 ms prior and 375 ms following the SES.

### ABR Recordings

ABR and behavioral recordings were acquired from each animal prior to and 2 hours following intraperitoneal (i.p.) injection of 250 mg/kg SS (concentration: 25 mg/mL 0.9% saline); post-SS tests were completed within 3 hours. Jastreboff et al. reported that after an i.p. injection of SS in rats, the maximum levels in blood serum occurred 1.5 hours later, while the maximum levels in the perilymph and spinal fluid reached maximum levels within 2–4 hours [32]. Animals were anesthetized before each ABR recording with ketamine (120 mg/kg) and xylazine (10 mg/kg) i.p., and respiration was monitored throughout to determine when additional supplemental doses were needed. Body temperature was kept constant at 37°C using a feedback-controlled heating pad (Physitemp TCAT2-LV Controller, Clifton, NJ).

Stimuli were generated digitally and controlled using a TDT RZ6 Multi-I/O Processor and acquired using BioSig software. Binaural acoustic signals were played through a multi-field (MF1) magnetic speaker (TDT, Alachua, FL) with a total harmonic distortion < = 1% from 1 kHz to 50 kHz, centered 0° azimuth in regards to the animal at a distance of 10 cm from the ear pinna. Tone bursts were presented at frequencies of 6, 12, 16, 20, 24, and 36 kHz (3 ms duration, 1 ms rise/fall time, alternating polarity) at a rate of 29 per second, attenuated in 5 dB steps from 80 dB SPL to 15 dB below threshold or 5 dB SPL, whichever was lower. Threshold was determined by visual inspection as the lowest intensity level which produced a defined peak in both replicates. GIN signals consisted of two 25 ms narrow band (1/6 octave) noise bursts centered at 6, 12, 16, 20, and 24 kHz, separated by nine increasing gap sizes ranging from no gap to 50 ms. Post-SS recordings were made with all GIN signals at 70 dB SPL, and pre-SS recordings were made at both 70 dB and 50 dB SPL for reference comparison to account for the approximately 20 dB increase in hearing threshold. All signals were calibrated using a Larsen Davis preamplifier, model 2221, with a 1/4” microphone and a Larson Davis CAL200 Precision Acoustic Calibrator (PCB Piezotronics, Inc., Depew, NY).

ABR recordings were acquired using a TDT RA4LI low-impedance digital headstage and RA4PA Medusa preamp with the active (noninverting) electrode inserted at the vertex, the reference (inverting) electrode below the right ear, and the ground electrode below the left ear. The responses were amplified (20x), filtered (300 Hz—3 kHz), and averaged using BioSig software and the System III hardware (TDT) data-acquisition system. A total of 256 tone burst signal and 150 GIN signal recordings were replicated for each acquisition, and muscle artifacts exceeding 7uV were rejected from the averaged response. All recordings took place in a soundproof booth lined with echo-attenuating acoustic foam.

### Data processing and statistical analysis

**Pre-Pulse Inhibition.** For behavioral assays, peak amplitude was defined as the largest positive voltage measurement within the post-stimulus recording window, and latency as the time where this occurred (before 100 ms in every recorded trial). If the peak amplitude measurement was below the mean RMS baseline measurement plus one standard deviation (taken during 125 ms of the animal’s movement in a no noise environment) then that trial (amplitude and latency) was discarded. Subsequently, the data were submitted to the general extreme Studentized deviate (gESD) test for outliers [33] where large extremes in the movement epochs were removed. This test was set to use α = 0.05 and a maximum outlier allowance equal to 5% of trials, although typically less than 2% of trials were removed. This outlier removal procedure was developed as a remedy to the occasional extreme fluctuations seen in behavioral assays, detailed in a review of the optimization of tinnitus behavioral assays by Longenecker and Galazyuk, where they recommend a statistical test for identification of outliers as an alternative to the common practice of removing the largest and smallest data points [16]. The gESD test is an improved iterative version of their recommended procedure, Grubb’s outlier identification test. The removal of less than 2% of trials is also consistent with their reported results in the removal of outliers [16]. The remaining trials were then averaged for each unique stimuli condition and the % PPI for each frequency was calculated as$100\%\times \left(1-\left(\frac{gap}{no-gap}\right)\right)$. All analysis procedures were built into a custom automated MATLAB program. The best three (of six) trials were averaged for the behavioral testing to give one data point (amplitude and latency) per animal for both the pre-SS and post-SS conditions.

**Auditory Brainstem Response.** For each ABR, peak to trough amplitudes were measured, and latencies computed as the time elapsed between the stimulus onset and each analyzed peak. These measurements were automated on a waveform-by-waveform basis using custom designed MATLAB software, visually verified by individual inspection, and corrected if necessary. Corrections were performed to ensure that the chosen peak was the last point before the negative slope, consistent with a fundamental method described by Hall [34]. After analysis the duplicate recordings for each signal parameter were averaged for each animal before further analytical analysis within GraphPad Prism version 6.01 for Windows (GraphPad Software, La Jolla, CA). This analysis included comparing the recovery of peak amplitudes and latencies in the second noise burst (NB2) waveform response to that of the first noise burst (NB1) response (100% x NB2/NB1). It also included the comparison of data taken in response to both equal intensity in SPL, as well as sensory level (SL) based on the average shift in threshold of each peak following SS. Grand averages were generated by cropping each waveform at the onset and offset of P1 and P5, respectively, and then aligning P1 of each waveform before averaging, with the thickness of the line indicating standard error of the mean (SEM).

**Statistics.** The statistical analysis and graphs were created using GraphPad, with graphed results presented as the mean ± SEM, except for the box and whiskers plot, which is displayed as a Tukey analysis. A repeated measures one-way ANOVA and Tukey’s multiple comparisons procedure were used to evaluate the effects of SS for each frequency across the two pre-SS and one post-SS conditions for the noise burst recovery in ABR GIN testing. A two-way ANOVA and Sidak’s method for multiple comparisons was used to evaluate the effects of the NB (1 versus 2) and gap duration for pre- and post-SS ABR GIN conditions. These two statistical methods were also used to evaluate behavioral testing and ABR amplitudes and latencies (salicylate x frequency), as these tests were expected to show effects of salicylate treatment across all frequencies and intensities, unlike GIN recovery measurements. A paired t-test was used to compare behavioral response latencies between the gap and no-gap trials before and after SS induced tinnitus.

## Results

### Behavioral Assays

**Fig 1:**
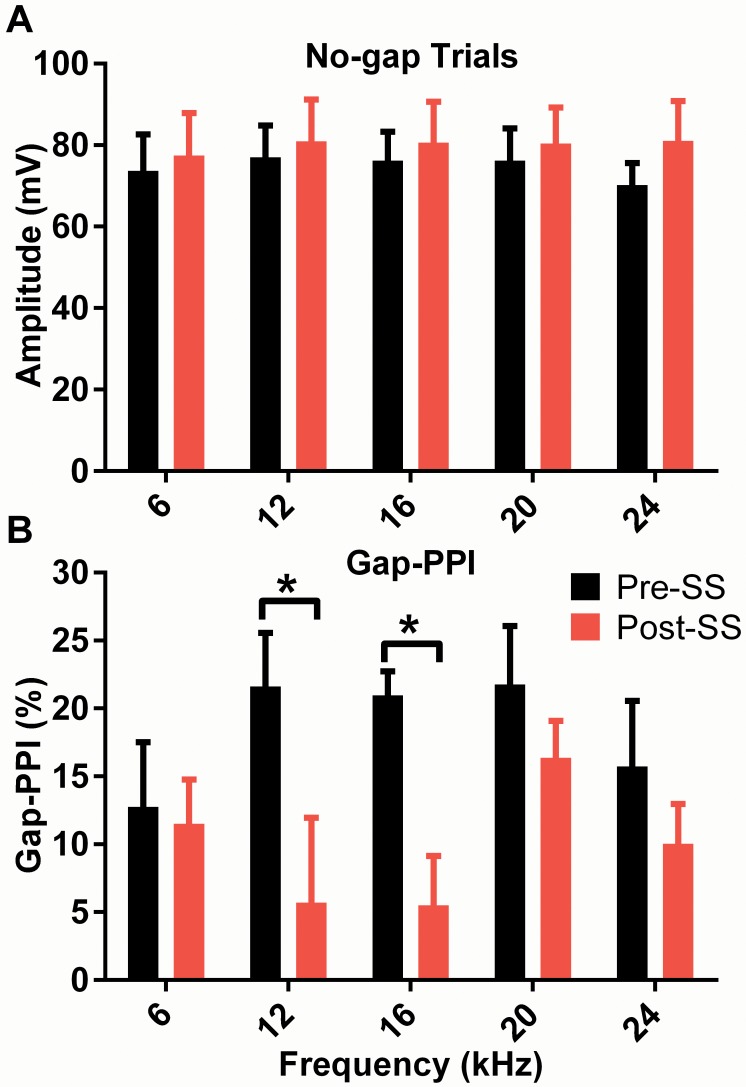
Behavioral manifestation of tinnitus on startle amplitude (A) and narrow band gap-PPI (B) measurements. (A) Maximum amplitude of the startle response to the SES with the (no-gap) carrier is shown as a function of the carrier center frequency. Although no significant effects were observed, a trend towards an increase in the magnitude of the ASR was noted at all frequencies following induction of tinnitus with salicylate. (B) The induction of perceptual tinnitus is indicated by significantly reduced gap-PPI (multiple comparisons, *p*<0.05) at the predicted tinnitus frequencies, the 12 and 16 kHz gap carriers.

Our results show no significant effect of SS on the magnitude of the ASR amplitude post-SS (Fig. 1A) presented without gaps. This is fortuitous because a stable ASR indicates that the PPI method is not compromised by SS-related changes in the base ASR. This result also lends confidence to the finding that SS had a significant effect on gap-PPI. Prior to SS-induced tinnitus, a 50 ms gap in a 70 dB SPL narrow-band noise of varying center frequencies reduced the startle response by approximately 19% (varying from 13% to 22% for individual frequencies) compared to the carrier with no gap condition. Though SS had a significant effect on gap-PPI, *F*(1,90) = 11.8, *p* = 0.0009, n = 10, neither frequency nor an interaction of the two was found to contribute to the variance. We found behavioral evidence of tinnitus in mice following SS, as observed in reduced PPI at 12 and 16 kHz, a significant decrease of 15.9 ± 7.4% PPI at 12 kHz and 15.4 ± 4.0% PPI at 16 kHz compared to baseline measurements (Fig. 1B). Results were consistent with the premise that tinnitus fills in the gap and reduces the salience of the silent interval. SS did not have significant effects on carriers of the other frequencies. Subsequently we also we also tested the effects of a broadband noise (BBN) gap carrier following SS-induction of tinnitus in 3 different animals (S1 Fig.), with the 16 kHz narrow band noise carrier for comparison, and found that gap detection was not significantly affected for the BBN carrier while detection to the 16 kHz carrier was again significantly reduced. These results show that while the startle amplitude remains unaffected by drug-induced tinnitus, the perception of large silent gaps is significantly impaired in the 12–16 kHz range.

The latency at which the highest positive voltage (i.e. amplitude) of the startle response occurred for both the no-gap and gap trials was averaged across the 3 trials for each mouse, and then across the group (n = 10). The ASR latency for the trials containing a 50 ms gap was significantly affected by SS-induced tinnitus (though not frequency specific), *F*(1,90) = 11.9, *p* = 0.0009, with a mean latency of 45.1 ms pre-SS, and 38.8 ms post-SS. There was no significant effect of SS on the latency of no-gap trials, though a decrease from an average latency of 42.4 ms pre-SS to 38.6 ms post-SS was observed. Prior to administering SS, there was a difference of 2.7 ms (t = 2.3, df = 49, *p* = 0.0276) between the latency of the gap and no-gap trial, however, following SS no latency difference existed. The latency data support hyperexcitability in the neural circuit eliciting the response to a gap carrier signal.

### ABR Recordings: Tone Burst Stimuli

**Fig 2:**
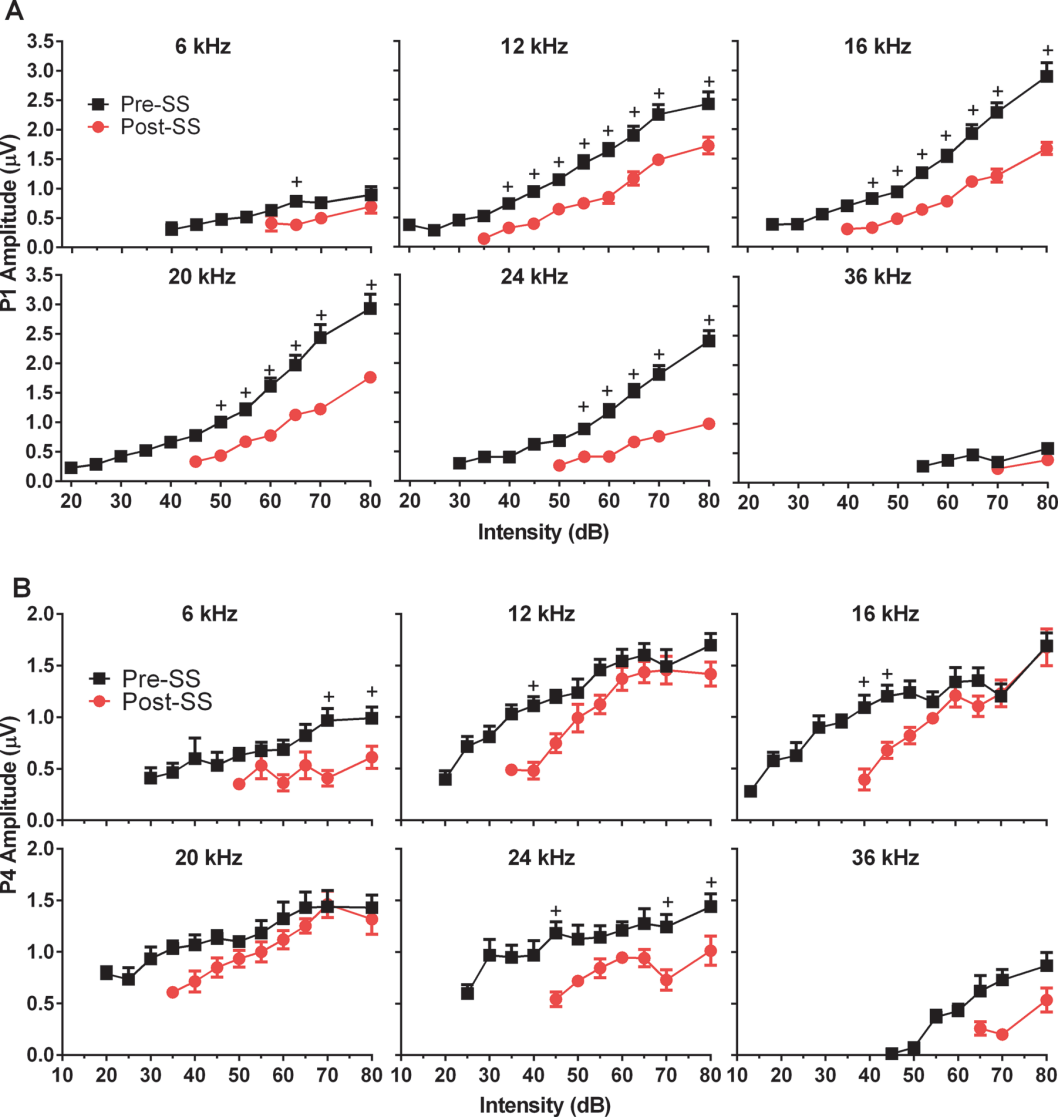
Physiological effects following induction of tinnitus on ABR P1 and P4 peak amplitudes. (A) SS-induced tinnitus significantly decreased P1 amplitudes at all frequencies. (B) P4 midbrain responses show significantly reduced amplitudes at all frequencies. Note that P4 amplitudes approximate control amplitudes at higher intensities for frequencies associated with tinnitus, while P1 amplitudes remain depressed. Multiple comparisons tests showed that only lower intensities were significantly decreased for 12, 16, and 20 kHz (+ = significance by Sidak’s multiple comparisons method).

ABR thresholds exhibited an average threshold increase following SS injection of 21 ± 1.6 dB SPL, *F*(2,150) = 293, *p*<0.0001, n = 10, which was significant for all frequencies in post hoc testing (*p*<0.0001). The highest increase in threshold, averaging 24.5 dB SPL, occurred in response to 16 kHz tones, and the lowest average increase of 16 dB SPL was in the 36 kHz ABR responses. The ABR threshold shift as a function of frequency can be visualized in S2 Fig. These thresholds were determined as the lowest intensity which elicited a replicable peak in the waveform response, note that this replicable peak may not have been one of those analyzed and therefore may be different than the P1 or P4 thresholds (indicated by the lack of response) shown in Fig. 2.

To assess the effects of SS on the peripheral and central auditory system, the latency and amplitude of P1, P2, and P4 were examined in the responses to short duration tone bursts. We found that SS significantly affected P1 amplitude (Fig. 2A) for all frequencies; 6kHz: *F*(1,52) = 15, *p* = 0.0003; 12kHz: *F*(1,104) = 99, *p*<0.0001; 16kHz: *F*(1,105) = 141, *p*<0.0001; 20kHz: *F*(1,96) = 146, *p*<0.0001; 24kHz: *F*(1,78) = 171, *p*<0.0001; 36kHz: *F*(1,23) = 6 *p* = 0.025; n = 8. Significance of multiple comparisons test methods is indicated in Fig. 2A and applies to all graphed amplitudes and latencies. There was a significant main effect of SS on P2 amplitude (not shown) at only 6 kHz, *F*(1,64) = 5, *p* = 0.03, and 24 kHz, *F*(1,87) = 13, *p* = 0.0004. Post-hoc testing revealed that P2 amplitudes were significantly reduced only at the 80 dB presentation level for both frequencies (6k: *p* = 0.0003, 24k: *p* = 0.0374). There was no overall effect for P2 amplitude at 12, 16 and 20 kHz, but mean P2 amplitudes were increased at 80 dB for 12, 16, and 20 kHz. There was a significant effect of SS on P4 amplitude at all frequencies; 6kHz: F(1,59) = 21, *p*<0.0001; 12kHz: F(1,108) = 25, *p*<0.0001; 16kHz: F(1,107) = 20, *p*<0.0001; 20kHz: F(1,107) = 9, *p* = 0.0029; 24kHz: F(1,92) = 47, *p*<0.0001; 36kHz: F(1,29) = 10, *p* = 0.0032; n = 8. P4 amplitudes elicited by low intensity tone bursts were generally reduced across all frequencies post-SS (Fig. 2B); however, this reduction was minimized at higher intensities. For frequencies of 12, 16, and 20 kHz P4 amplitudes varied in a complex manner as a function of intensity, where amplitude was significantly reduced for lower intensities, but as intensity increased there was an abnormal increase of P4 amplitude, suggestive of recruitment.

**Fig 3:**
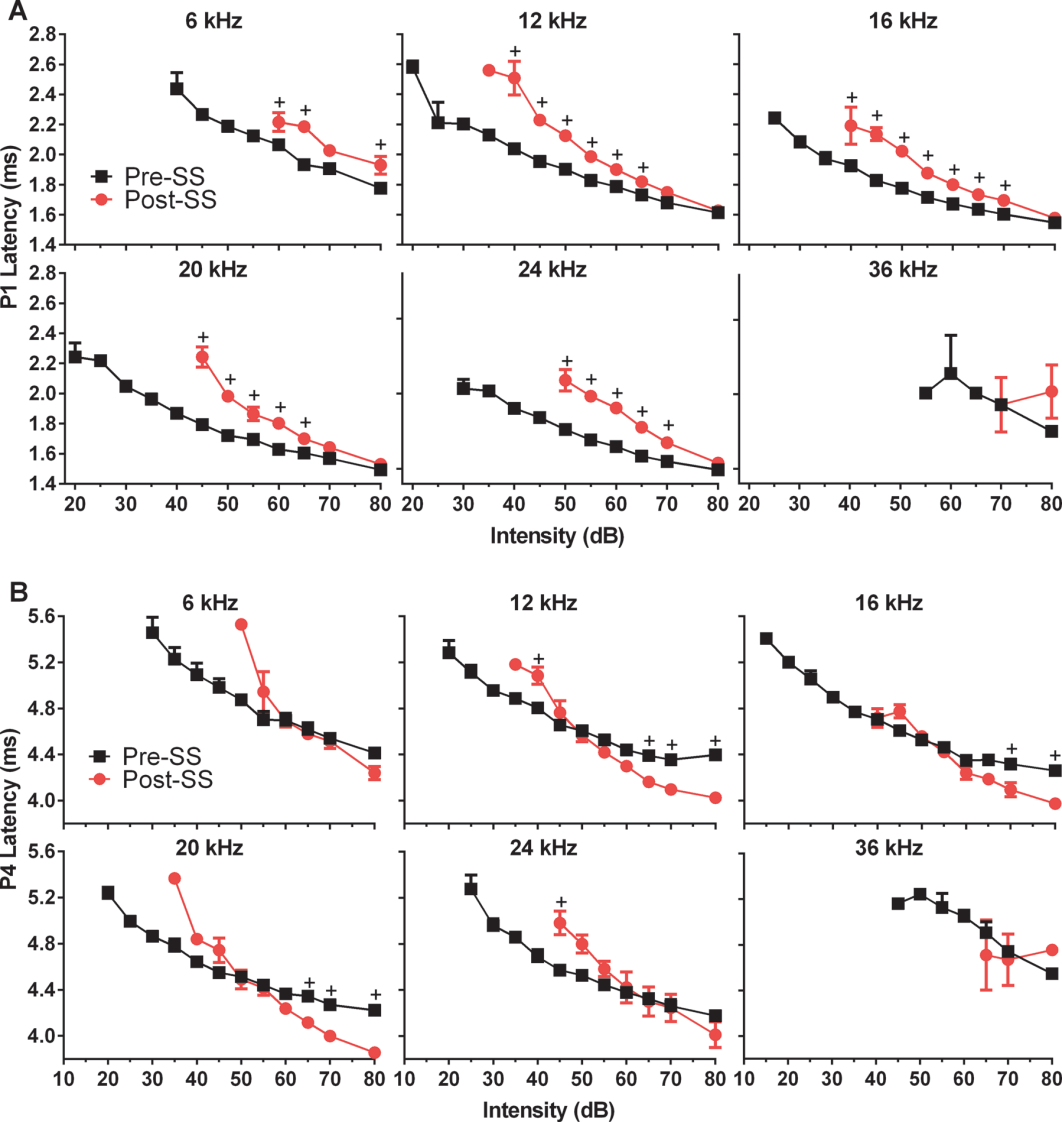
Effects of tinnitus induction following SS on P1 and P4 ABR peak latencies. (A) SS significantly increased P1 latencies as a function of stimulus intensity for all test frequencies except 36 kHz. Note that latencies converge to control values at higher intensities. (B) SS significantly affected P4 latencies in a complex manner for all test frequencies except 6 and 36 kHz. SS decreased the latency for P4 at higher intensities, but not at lower intensities. This effect on P4 was also seen in the GIN testing. (+ = significance by Sidak’s multiple comparisons method).

P1 latencies were significantly increased for all frequencies except 36 kHz following SS treatment; 6kHz: *F*(1,52) = 45, 12kHz: *F*(1,104) = 308, 16kHz: *F*(1,105) = 209, 20kHz: *F*(1,96) = 166, 24kHz: *F*(1,78) = 262; *p*<0.0001 for all, n = 8; although they were not affected at high intensities (Fig. 3A). P2 latency (not shown) demonstrated the same effects; 6kHz: *F*(1,49) = 8, *p* = 0.0066; 12kHz: *F*(1,96) = 27, *p*<0.0001; 16kHz: *F*(1,92) = 88, *p*<0.0001; 20kHz: *F*(1,98) = 126, *p*<0.0001; 24kHz: *F*(1,79) = 103, *p*<0.0001; n = 8. SS also had a significant effect on P4 latency; 12k: *F*(1,108) = 17, *p*<0.0001; 16k: *F*(1,107) = 10, *p* = 0.0019; 20k: *F*(1,106) = 11, *p* = 0.0015; 24k: *F*(1,92) = 5, *p* = 0.0348; n = 8. This resulted in a complex interaction (Fig. 3B) where P4 latency which was prolonged in trials following tinnitus induction, compared to control trials for lower intensities, and then decreased at higher intensities where amplitude was also noted to grow rapidly.

Since P1 and P2 amplitudes and latencies demonstrated different effects at the highest intensity levels, we compared the 80 dB SPL responses following SS-induced tinnitus to the equal SL pre-SS response. No significant effect was seen for P1 or P4 amplitude. When we examined P4 amplitude at the tinnitus frequency (16 kHz) there was a significant increase in P4 amplitude following SS, of 0.53 μV (t = 2.5, df = 7, *p* = 0.0496). There was also a significant decrease for both P1 (*F*(1,79) = 13.7, *p* = 0.0004) and P2 latency (*F*(1,80) = 100, *p*<0.0001) following SS induced tinnitus. This was an average of 0.16 ms for P1 latency post-SS, though post hoc testing did not reveal a significant effect for any frequency specific responses. Significant post hoc decreases were seen for P2 latencies in response to all frequency tone bursts, averaging 0.25 ms, except for 36 kHz where latency was unchanged. At 80 dB SPL, P4 latency was reduced and P2 amplitude was increased, making equal SL comparisons unnecessary.

**Fig 4:**
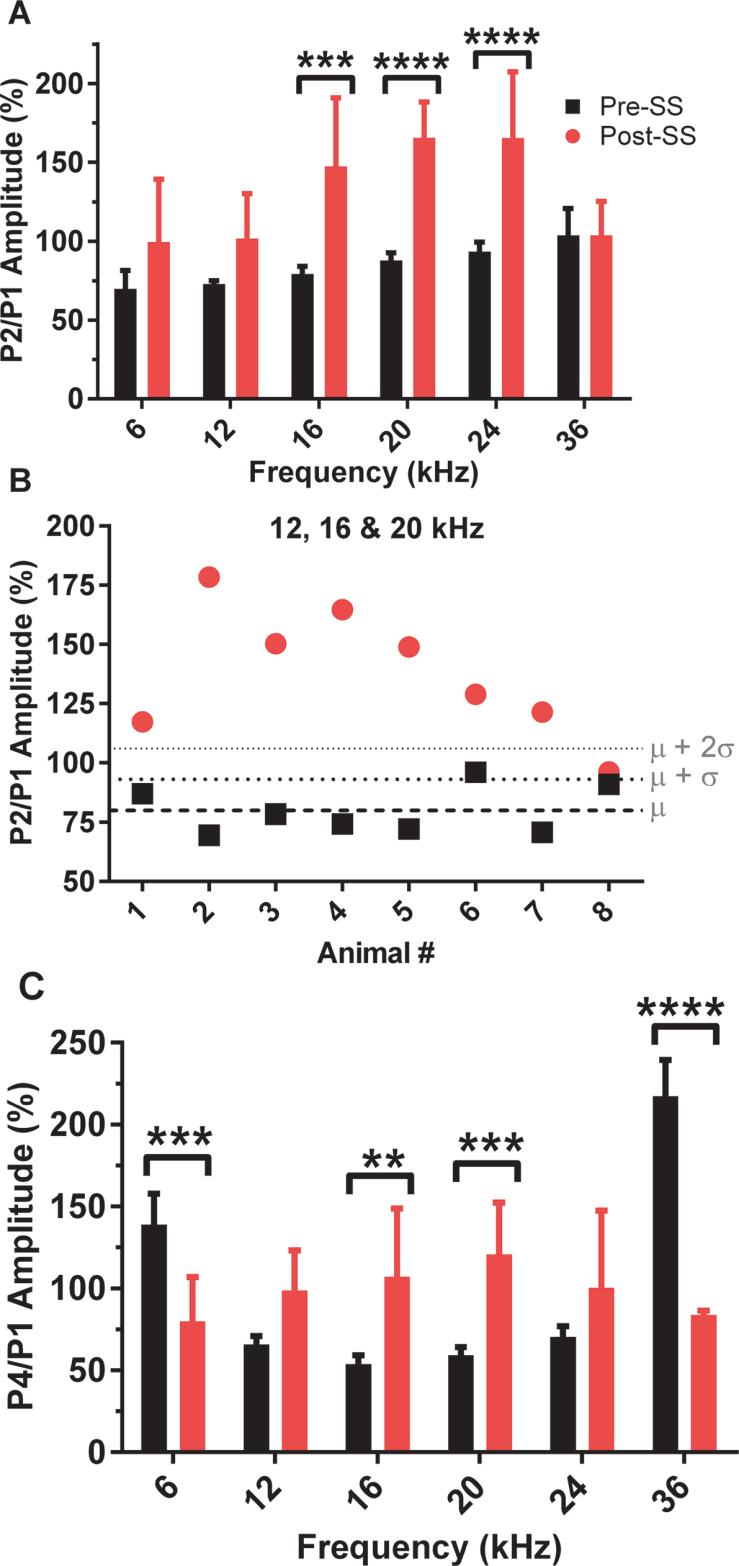
Induction of tinnitus following SS alters the P2/P1 and P4/P1 amplitude ratios in response to 70 dB tone bursts. (A) SS increased the P2/P1 amplitude ratio in response to all tone burst frequencies. (B) Each animal demonstrated a significant increase in the P2/P1 amplitude ratio for tinnitus frequencies (12, 16, & 20 kHz) following SS-induced tinnitus, confirming that the P2/P1 ratio is a sensitive measure of tinnitus. Dashed lines indicate the mean, mean + 1 standard deviation (SD), and the mean + 2 SDs for baseline testing. (C) SS-induced tinnitus also significantly affected the P4/P1 amplitude ratio; multiple comparisons analysis was significant for the increases in response to 16 and 20 kHz tones, while significantly decreased for 6 and 36 kHz responses (multiple comparisons test: **p <0.01, ***p<0.001, ****p<0.0001).

In order to determine if there was a predictive effect of tinnitus on P1, P2, and P4 amplitudes, we calculated the P4/P1 and P2/P1 amplitude ratio of the responses to the 70 dB SPL tone bursts at each frequency (Fig. 4A and 4C). Similarly, we plotted the P2/P1 ratio in response to tones in the predicted tinnitus range (12, 16, and 20 kHz) for each individual animal before and after salicylate induced tinnitus (Fig. 4B). Results demonstrated that this analysis can be used as a reliable indicator of tinnitus in a single animal over one ABR session. The group analysis revealed an effect of SS on the P2/P1 amplitude ratio, *F*(1,78) = 42.3, *p*<0.0001, n = 8, significant at presentation tones of 16, 20, and 24 kHz (increases of 68 ± 16%, 78 ± 9%, and 72 ± 16% respectively). The P4/P1 amplitude ratio was also significantly increased, *F*(1,80) = 102, *p*<0.0001, n = 8, with post hoc significance in response to tones of 16 and 20 kHz, corresponding to 53 ± 16% and 61 ± 12%, respectively. The opposite effect occurred for the P4/P1 ratio responses to tones presented outside of the tinnitus frequencies, with a significant decrease at 6 and 36 kHz of 59 ± 21% and 134 ± 22%.

We also examined the correlation between the behavioral measures of tinnitus (gap-PPI reduction) and the ABR measures of tinnitus. No significant correlation was seen between the reductions in gap-PPI and the NB2/NB1 recovery (discussed below) or the increase in P4/P1 amplitude ratio; however, correlation of the P2/P1 and gap-PPI was significant for the tinnitus percept (16 kHz) only, *r* = -0.738, *p* = 0.0365. The linear regression for this correlation can be seen in S3 Fig., where the slope was equal to-4.0 ± 1.5 (R^2^ = 0.55), and differed significantly from zero (*F* = 7.18, *p* = 0.0365). We also no significant correlation between the ABR threshold shift and the behavioral and electrophysiological assays following tinnitus induction.

### ABR Recordings: Gaps-in-Noise

**Fig 5:**
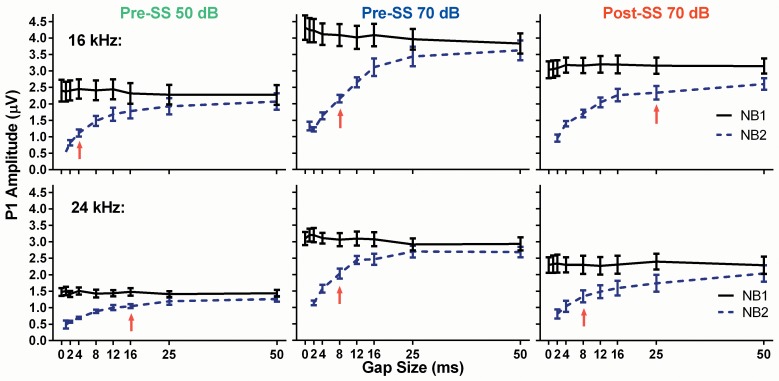
Comparison of P1 amplitude gap recovery functions for NB1 and NB2 as a function of increasing gap duration for 16 and 24 kHz. The solid lines represent the NB1 response and the dashed lines represent the NB2 response. Red arrows indicate the last gap size where NB1 and NB2 amplitudes were significantly different following multiple comparisons tests. Recovery functions to 16 and 24 kHz NBs were shown because 16 kHz is the expected tinnitus frequency and 24 kHz was not expected to match the pitch of tinnitus for any animal.

**Fig 6:**
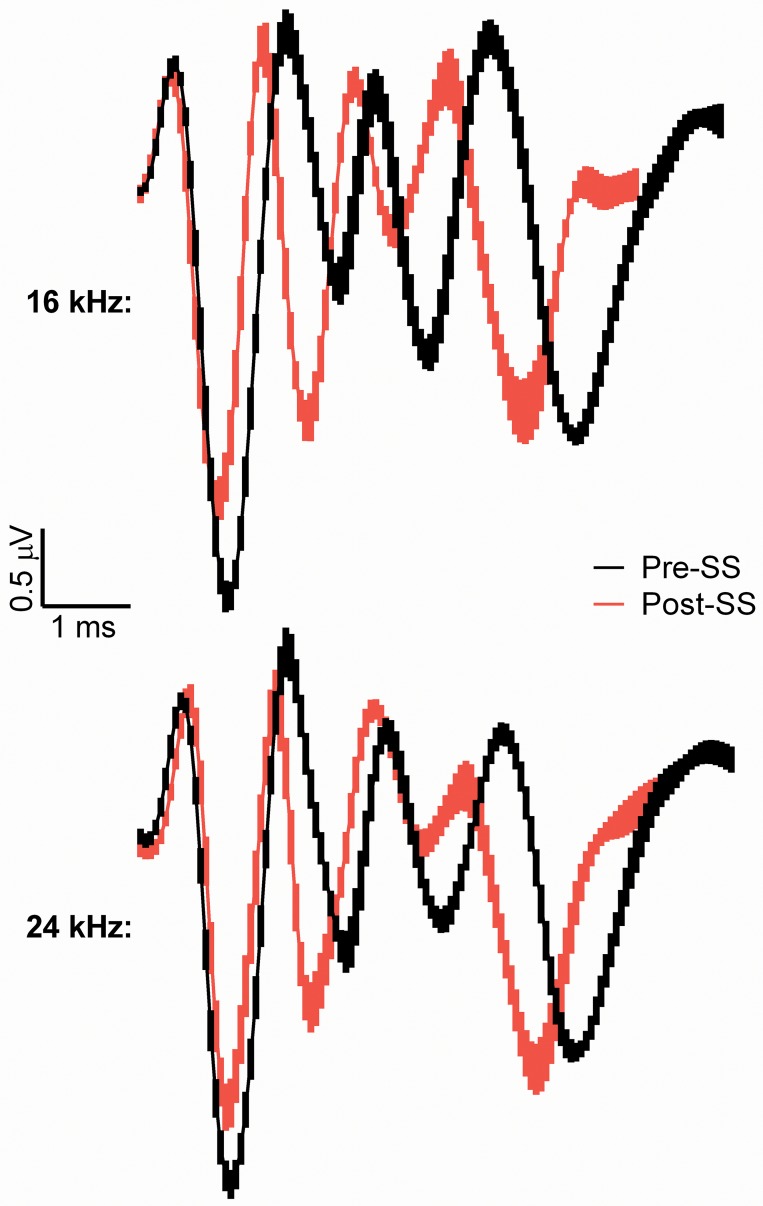
Grand average ABR waveforms (N = 8) prior to (black) and following (red) SS-induced tinnitus and elicited by the second 70 dB SPL noise burst (NB2) following a 50 ms silent gap. NB2 grand averages are shown to the 16 and 24 kHz stimuli. Waveforms were aligned along P1 before averaging, and SEM is shown as the thickness of the line. SS significantly decreased the peak to trough amplitude of P1 for 16 kHz but not 24 kHz (shown in Fig. 8), and significantly decreased the latency of P4 (shown in Fig. 9).

**Fig 7:**
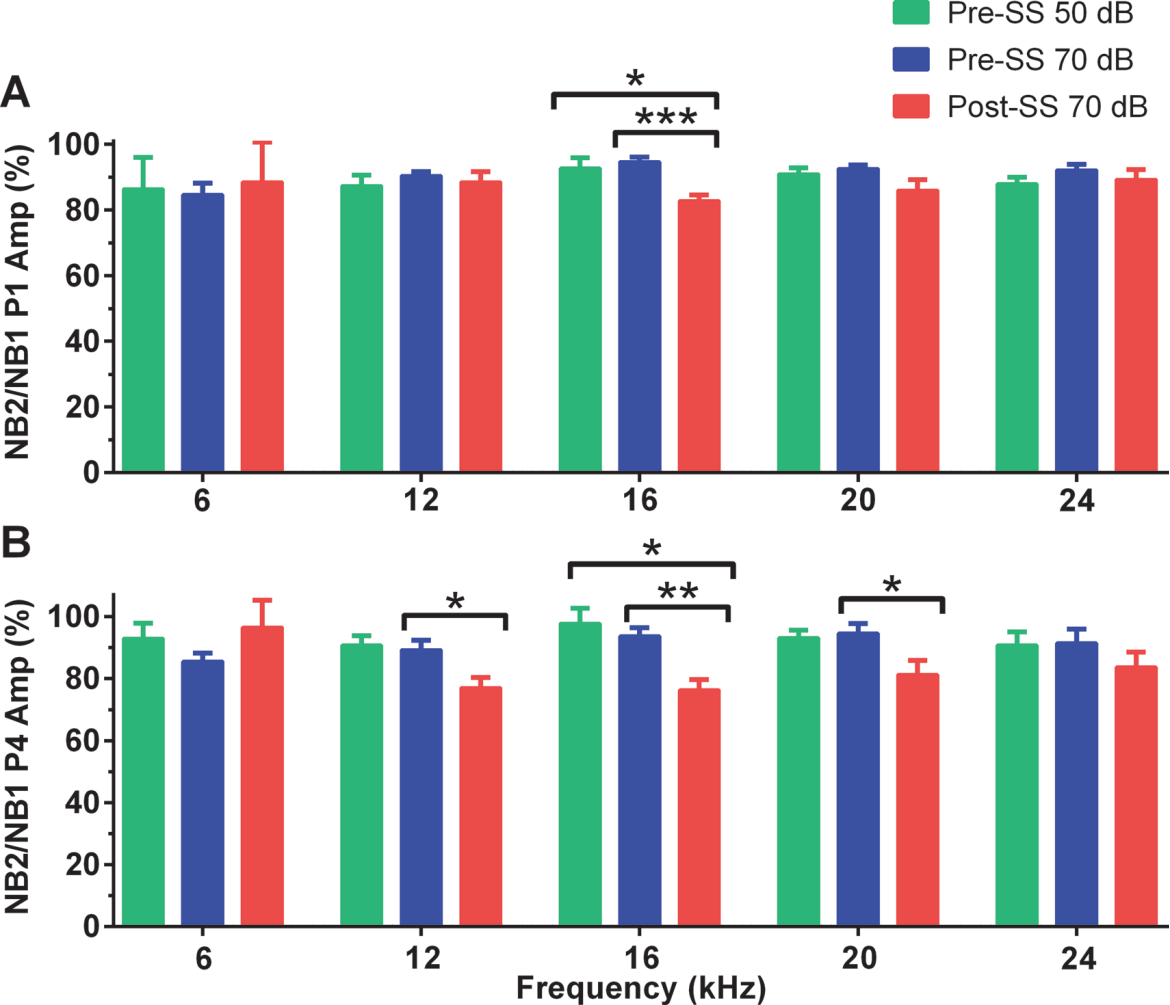
SS-induced effects on the NB2/NB1 ratio of P1 amplitude. (A) Following tinnitus induction the mean NB2/NB1 P1 amplitude ratio was significantly reduced only for 16 kHz, when compared to both pre-SS intensity conditions. Note that a 100% amplitude ratio indicates that the amplitudes elicited by NB1 and NB2 were equal. (B) P4 NB2/NB1 amplitude ratios were significantly decreased for 12, 16, and 20 kHz when compared to the 70 dB pre-SS condition. Only at 16 kHz did the post-SS ratio significantly decrease compared to the pre-SS 50 dB test (multiple comparisons significance: *p<0.05, **p <0.01; ***p <0.01).

The GIN ABR testing was devised as a parallel electrophysiological method to the gap-PPI behavioral assay, consistent with the theory that the tinnitus percept “fills in” the silent gap when the frequency of gap carrier matches the pitch of the tinnitus. Here we presented two 25 ms narrowband noise bursts separated by varying gap sizes, with the 50 ms gap used to determine the presence of tinnitus. Fig. 5 shows gap recovery functions (NB1/NB2) for the two pre-SS trials (green and blue) compared to post-SS (red) trials for the 16 and 24 kHz NB frequencies. The amplitude of P1 to NB1 is constant across all gap durations for all conditions, as expected, while the amplitude of P1 to NB2 increases systematically with longer gap durations. As expected, there was a main effect on P1 amplitude elicited by NB1 or NB2 for 16 kHz (Pre-SS 50dB: *F*(1,153) = 36, Pre-SS 70dB: *F*(1,154) = 102, Post-SS: *F*(1,153) = 33, *p*<0.0001 for all) and 24 kHz: (Pre-SS 50dB: *F*(1,98) = 112, Pre-SS 70dB: *F*(1,98) = 97, Post-SS: *F*(1,98) = 49, *p*<0.0001 for all). Post-SS, the NB2 response to 24 kHz centered noise bursts exhibits a slower recovery than the pre-SS functions; however, the recovery was faster than that of the 16 kHz (predicted tinnitus frequency) NB2 response, indicated by the lack of significant differences in multiple comparisons testing, denoted as red arrows in Fig. 5. Fig. 6 illustrates the grand averages for the NB2 pre-SS and post-SS responses to the 70 dB SPL 50 ms gap for center frequencies of 16 and 24 kHz. When the P1 amplitude for the NB2 response was calculated as a percentage of the NB1 response, which serves as a metric of neural recovery, a significant difference was only observed in the tinnitus region (16 kHz centered noise bursts), where there was a prolonged recovery following tinnitus induction. This recovery ratio, in response to the 50 ms gap (Fig. 7A), was significantly reduced (*F*(1.37,15) = 7.6, *p* = 0.0097, n = 12) post-SS by 9.9 ± 3.3% when compared to the 50 dB pre-SS condition and 11.8 ± 2.1% when compared to the 70 dB pre-SS condition. The change in the P4 NB2/NB1 amplitude ratio (Fig. 7B) was not as frequency specific, with significant slowing at 12, 16, and 20 kHz: (12k: *F*(1.56,16) = 6.6, *p* = 0.0118; 16k: *F*(1.38,15) = 7.9, *p* = 0.0083; 20k: *F*(1.85,20) = 4.8, *p* = 0.0215; n = 12). The recovery of the P2 amplitude NB2/NB1 ratio was not significantly different for any frequency/intensity condition.

The P1, P2, and P4 amplitudes in response to NB1 and NB2 were also analyzed to determine the effects of tinnitus on peripheral and central auditory structures. A two-way ANOVA indicated that SS significantly affected P1 (NB1: *F*(2,140) = 37, NB2: *F*(2,140) = 46), P2 (NB1: *F*(2,135) = 21, NB2: *F*(2,135) = 28), and P4 (NB1: *F*(2,140) = 20, NB2: *F*(2,140) = 12) amplitudes (*p*<0.0001 and n = 12 for all). Post hoc testing indicated that only P2 amplitude significantly increased post-SS in comparison to the 70 dB pre-SS condition; this was in response to both NB1 and NB2 at 16 and 20 kHz (NB1: *p* = 0.0003 and 0.0384, NB2: *p*<0.0001 and *p* = 0.0005, respectively). Average P4 amplitude also increased following SS for the NB1 response only at 16 and 20 kHz, although this increase was not significant. P1 amplitudes decreased following SS at all frequencies for both NB responses, indicating reduced excitatory drive.

**Fig 8:**
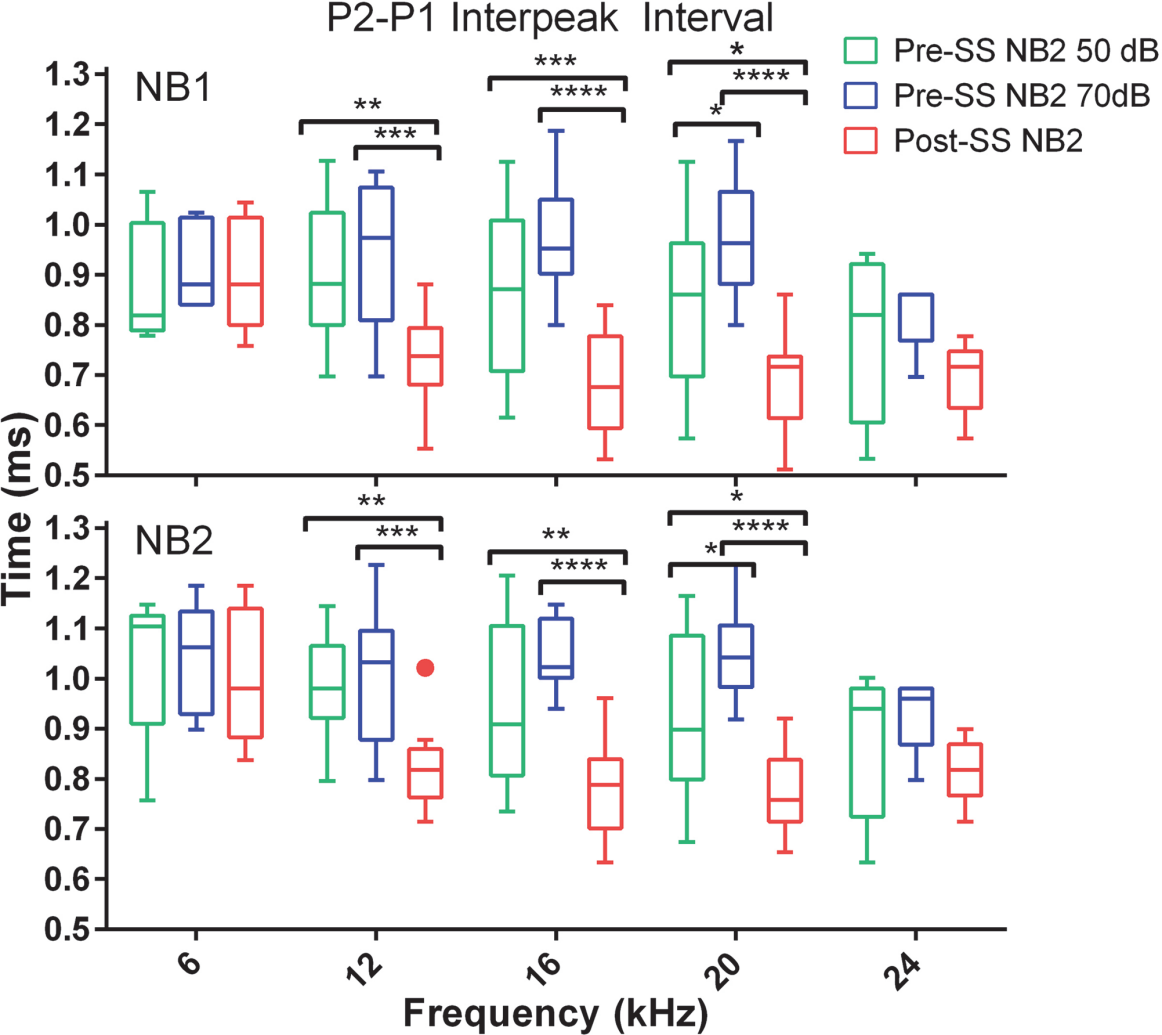
SS-induced tinnitus significantly decreased the ABR P2-P1 interpeak latencies elicited by NB1 (top) and NB2 (bottom) GIN frequency speficic noise bursts. Box plots show the median and upper and lower quartiles for the 50 dB pre-tinnitus (green), 70 dB pre-tinnitus (blue), and post-tinnitus (red) interpeak measures for the 50 ms gap condition (multiple comparisons test: *p<0.05, **p<0.01, ***p<0.001, ****p<0.0001).

**Fig 9:**
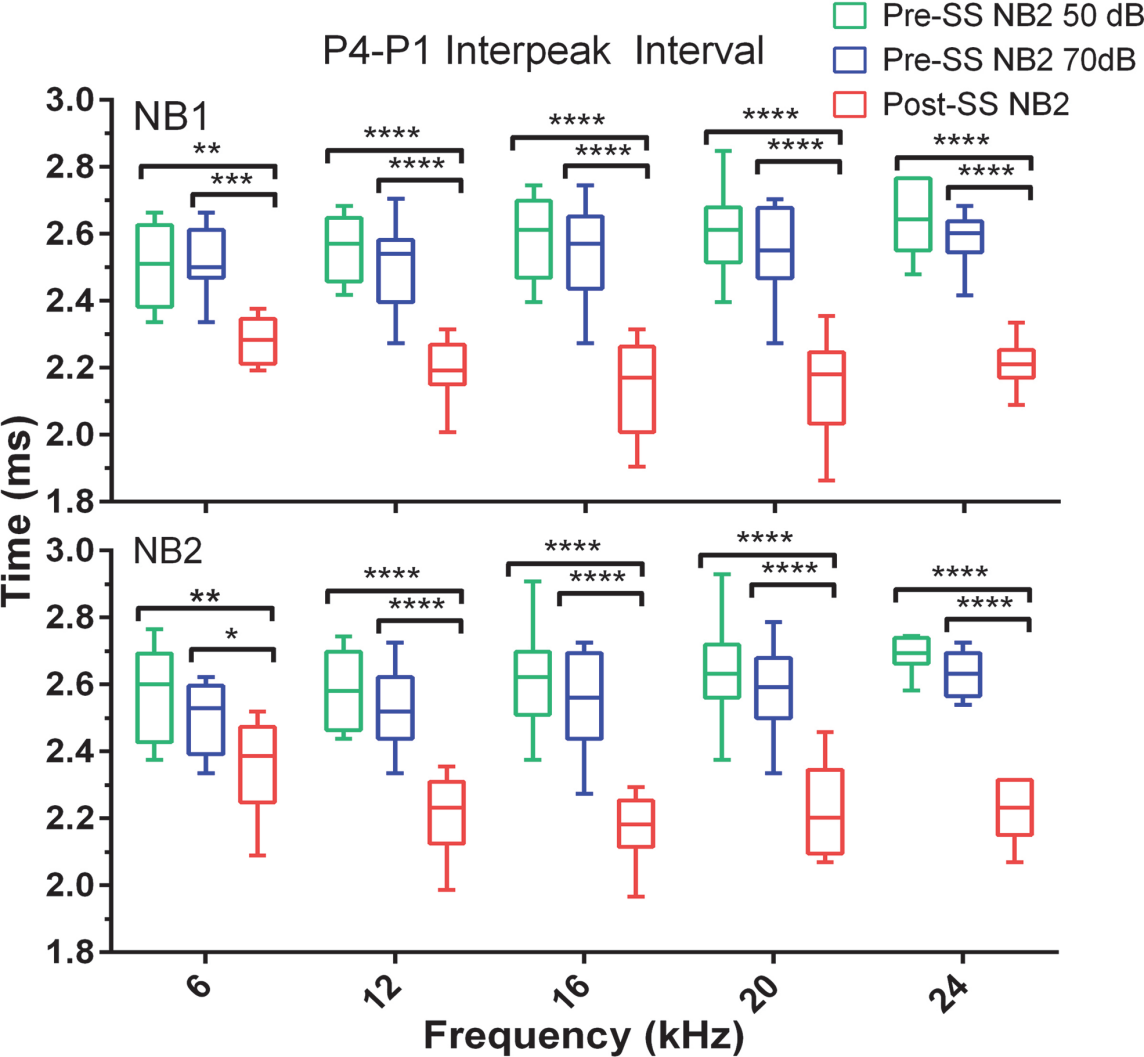
SS-induced tinnitus significantly decreased the ABR P4-P1 interpeak latencies to both NB1 and NB2 GIN noise bursts. The data is displayed identical to Fig. 8. Note that the NB2 response latency shown here is that following the 50 ms gap (multiple comparisons test: *p<0.05, **p<0.01, ***p<0.001, ****p<0.0001; n = 12).

P1 latency of both NB1 and NB2 responses was not significantly affected by SS when compared to the pre-SS 70 dB condition. However, P2 and P4 latency were significantly decreased following salicylate treatment when compared to both baseline conditions. The average post-SS P2 latency decrease (for all frequencies) compared to the 50 dB condition was 0.28 ± 0.02 ms for NB1 and 0.21 ± 0.06 ms NB2; for the 70 dB comparison there was a decrease of 0.26 ± 0.02 ms for NB1 and 0.18 ± 0.05 ms for NB2 following SS. The average post-SS P4 decrease (for all frequencies) compared to the 50 dB condition was 0.5 ± 0.03 ms for both NB1 and NB2; for the 70 dB condition there was a decrease of 0.34 ± 0.04 ms for NB1 and 0.30 ± 0.05 ms for NB2. As shown in Figs. 8 and 9, there were also significant post-SS decreases of the inter-peak intervals (compared to P1) for P2: (NB1: *F*(2,117) = 22, NB2: *F*(2,118) = 24) and P4: (NB1: *F*(2,139) = 154, NB2: *F*(2,139) = 136), *p*<0.0001. These results are also observed in the grand averages of ABR waves shown in Fig. 6 and indicate faster transmission times in the auditory brainstem after SS-induced tinnitus.

## Discussion

Currently, the clinical diagnosis of tinnitus in human patients relies on subjective measures such as self-reports, questionnaires, visual analog rating scales, and psychoacoustic matching [3,31]. However, many successful experiments have been performed which identify tinnitus in animal models based on the theory that the perceived phantom sound will distort the perception of silent gaps in narrowband noise. Unfortunately, the success of these test methods has not translated for clinical use with human subjects due to inconsistencies among the various test parameters. For example, Campolo and colleagues employed the GIN paradigm to assess tinnitus in human subjects, using a 50 ms gap imbedded in narrow band noise with a center frequency matching the tinnitus percept, and found that the method did not reliably differentiate tinnitus patients from control subjects. However, the authors suggest this may be due to tinnitus interfering with inhibition of the startle reflex but not discernment of gaps altogether [3]. A study comparing 20 tinnitus patients with 20 control subjects, all having normal hearing thresholds, found that the gap detection threshold (the shortest gap correctly identified 4 out of 6 times) was significantly higher for the tinnitus group [35]. Another study found that gap detection deficits were also seen in normal hearing human subjects suffering from tinnitus in comparison to controls, in response to both a low and high frequency gap carrier narrowband noise [31]. Therefore, it is likely that the ABR responses to GIN stimuli will demonstrate greater effects when tinnitus is present as compared to psychophysical testing in human subjects. While functional magnetic resonance imaging (fMRI) is one technique used to measure neural activity in the effort to determine functional changes in the auditory midbrain of tinnitus sufferers, fMRI lacks the temporal sensitivity with respect to the degree of neural synchronization on a millisecond timescale [27], it is more expensive, and time consuming [3]. Auditory evoked potentials examining the middle latency response and steady state response have also proven promising, though results have also remained inconsistent and difficult to interpret [36]. As the ABR is an inexpensive and commonly used clinical measurement, this method will prove especially useful in tinnitus diagnosis if translatable.

We used pre-pulse inhibition of narrowband GIN carriers as the behavioral assay to determine if SS-induced tinnitus in CBA/CaJ mice would be consistent with those reported in the literature. Previous studies associated the loss of salience to large silent gaps embedded in narrowband noise carriers in the tinnitus region as evidence of perceptual tinnitus at those frequencies [21,22]. Based on this hypothesis, our results indicated that CBA/CaJ mice were experiencing tinnitus in the 12–16 kHz range following administration of SS (Fig. 1). We found that detection of tinnitus using ABR P1 amplitude recovery functions was more specific than behavioral assays in regards to significant frequency related changes, as differences were only seen in responses to 16 kHz and not 12 kHz. We believe that the SS-induced hearing loss is not affecting the decrease in the 16 kHz recovery ratio, as the threshold shift from 6–24 kHz was consistent (within 3.4 dB), and the recovery was similar for both test intensities prior to SS-induced tinnitus. Although recovery of the P4 amplitude was impaired across a range of frequencies, this can also be viewed as an important indicator of tinnitus, as gap detection in the IC has been shown to be independent of sensorineural hearing loss [37]. It is also important to note that this prolonged recovery was the result of SS-induced increases in P4 amplitude responses to the mid-frequency NB1s, and unchanged amplitudes in response to NB2. This is in contrast to changes in other ABR peaks, where decreased P1 amplitudes were observed (more so for mid-frequency NB2 responses) and P2 amplitudes increased, in response to both NBs. While this initially appears to contrast the work by Deng et al., which showed SS induced no changes in the amplitudes of IC evoked potential for input/output functions and reduced the amplitude of the IC evoked potential following gaps embedded in broadband noise [38], this was similar to our results for responses to stimuli outside of the tinnitus frequency.

A secondary goal of this study was to further investigate the neural correlates of tinnitus through the analysis of pure tone-evoked ABR peaks generated from the cochlea/AN, CN, and auditory midbrain. We found altered neural activity after tinnitus induction, specifically a decrease in the AN activity (P1) and an amplification of sound-evoked activity originating from the region of the CN and its connections, as observed by P2 amplitude enlargement and increases in the P2/P1 and P4/P1 amplitude ratio. While the mechanisms of SS-induced tinnitus have not been precisely determined, salicylate is known to reduce outer hair cell motility [29], which could reduce AN firing rate and synchrony, and therefore decrease the P1 amplitude. This is consistent with our results, which found SS significantly decreased P1 amplitude (Fig. 3A) to all test frequencies, and is in agreement with previous studies in rats which found reduced CAPs following salicylate administration [24,39]. Similar to our results, rats given a high dose of aspirin demonstrated reduced ABR P1 amplitudes at all test frequencies (2, 4, and 8 kHz) [23]. Many studies have implicated the CN in tinnitus generation, including spontaneous activity resembling that of tone evoked responses [40] and increases in stimulus evoked responses from fusiform cells of the dorsal cochlear nucleus (DCN) [41] following noise induced tinnitus. Similarly, tinnitus induction via noise exposure in guinea pigs resulted in changes in both the spontaneous and sound-evoked DCN activity, with an observed correlation between behavioral evidence of tinnitus and long-term somatosensory increases of sound-evoked responses [42]. Although discussions exist about whether the organization of the rodent DCN is dramatically different than that of humans and primates, a recent study found anatomical evidence that the use of studies in the rodent DCN can be validated [43]. Though the DCN is not a generator of the ABR, it projects to the IC, and therefore may still influence ABR responses [44]. The ventral cochlear nucleus (VCN) was recently shown to play a significant role in tinnitus perception, based on elevated sound-evoked activity originating in the pathway following this structure, after evaluating ABRs of human tinnitus sufferers and hearing matched controls [27]. Increases in the post-SS ABR P2 amplitude, along with the significantly reduced latency, suggest that the SS-induced tinnitus in our mouse model is related to hyperactivity in the CN. Similarly, the IC has also been implicated in tinnitus generation, and we found that P4 amplitude was affected in an intensity dependent manner. Though the amplitude was significantly reduced for lower intensities, there was an abnormal increase for higher intensities in responses to tones at the tinnitus frequencies of 12 and 16 kHz. Results examining the tone burst evoked local field potentials (LFPs) within the IC found that they were unchanged following SS, similar to the P4 amplitudes at high intensities [45].

A study of ABRs in human tinnitus sufferers found that the wave III/I and V/I amplitude ratios were elevated when compared to matched non-tinnitus patients [27]. This study agrees with our results, which demonstrated significant increases in the P2/P1 and P4/P1 amplitude ratios after SS-induced tinnitus. We also found a significant correlation between the increase of the P2/P1 ratio and reduced gap-PPI only at 16 kHz, following SS-induced tinnitus. This may provide an interesting index for future studies of noise-induced tinnitus, where setting a cutoff value to determine which mice are experiencing tinnitus is necessary. Reduction of wave I amplitudes in human subjects were attributed to many possibilities including the loss of inner hair cells, reduced excitability of auditory nerve fibers (ANFs), or degeneration of subsets of ANFs for tinnitus sufferers [27]. The difference in amplitude at the higher intensities of normal human ABRs without any distinctions between groups in response to lower intensities, corroborates with both our results showing larger decreases in P1 amplitude at high intensities, as well as the finding that a selective loss of high-threshold AN subtypes results in proportional decreases in amplitudes at suprathreshold levels [27,46]. Gu et al. also argues that ABR waves in humans subsequent to wave I originate in the VCN, and that the increased III/I and V/I ratios reflect increased activity in populations of the spherical bushy cells and their projections to the IC, especially when compared to the reduced activity of the AN [27].

In line with the theory that tinnitus relies on amplification of central neural pathways, changes in gross potentials following tinnitus induction from the IC and auditory cortex (AC), among other structures, have been reported previously. Following systemic SS injection, no change was seen in the amplitude or latency of the response from electrodes implanted in the IC of rats, indicating that the reduced output of the cochlea was already partially amplified when it reached this structure, though a threshold shift of approximately 20 dB SPL occurred [20,38,45]. The same procedure was also repeated for the AC, where the response amplitude was significantly enhanced to higher intensity sounds, and the threshold shift was consistent with the cochlea and IC increases of approximately 20 dB SPL. There were also shifts in the characteristic frequencies (CFs) of the AC responses, where neurons with CFs outside the 10 to 20 kHz range shifted into the area (10–20 kHz) of predicted tinnitus pitch. Direct application of SS to the AC also increased sound evoked activity [47], however when SS was applied to the cochlea, the amplitude of the AC LFPs was paradoxically reduced [20,45]. These findings indicate that acute doses of SS can directly affect the central auditory system, while reducing peripheral deficits and introducing tonotopic reorganization in central auditory structures [20,45,48].

Temporal processing is also significantly affected in tinnitus sufferers, as demonstrated by the reduced gap detection threshold seen in numerous human and animal studies [10,11,21,31,35]. These observations prompted our analysis of the ASR and ABR latencies, where we found significantly faster behavioral and electrophysiological response latency originating from the brainstem following tinnitus induction. Chen et al. found reduced reaction times in the ASR of rats following SS, which was hypothesized to be related to increased excitability within the central auditory pathway [49]. Comparatively, our results also indicated significantly reduced latency post-SS of the ASR for the trials containing a gap. This is likely not due to the hearing loss following SS, as it has been shown that ASR latency increased with age in C57 mice (which lose their hearing much earlier in life) but not in CBA mice, indicating that hearing loss is likely to cause increases in ASR latency [50]. The latency decrease may be associated to the similar reduction of the ABR NB2 P2 latency following a large gap, since the CN is a key auditory structure in the ASR circuit [12,51]. SS eliminated the latency difference between gap and no-gap trials by reducing that of the gap containing trial, indicating hyperexcitability in the circuit controlling inhibition of the ASR. This is consistent with research demonstrating that the IC plays a central role in mediating inhibition of the ASR [52], as well as our results showing reduced latency of the ABR P4 at high intensities. There was also a significant decrease in the latencies of P2 and P4 in the GIN ABR following tinnitus induction that occurred despite no significant change in P1 latency. In response to tone bursts, only P4 showed decreased latency at high intensities, however, when equal SL responses were compared to the highest intensity post-SS response latencies, P1 and P2 latencies were also found to be reduced. This is also consistent with findings of reduced P1 latency at equal sensation levels in chinchillas following acoustic trauma [53]. Coomber et al. found a reduction in latency of all measured ABR waves (II, IV, and V) in response to tonal stimuli that matched the center of the acoustic trauma frequency in guinea pigs with evidence of tinnitus [6]. Overall, our results reveal significant changes in the synchrony and firing of neurons in the brainstem, consistent with findings in other animal models.

The aim of this study was to establish a reliable and efficient method for verifying the presence of tinnitus in a mouse model. This was developed for both potential translation to clinical use, as well as for the study of prospective treatments of tinnitus in a mouse model. Collecting ABR responses to two different stimulus paradigms, a presentation of short duration tone bursts and a longer duration dual noise burst utilizing silent gaps, proved successful for the rapid identification of tinnitus in individual animals and the determination of the perceived tinnitus pitch. In summary, these results demonstrate the efficacy of a GIN ABR protocol that can increase the efficiency of studying tinnitus in animals, as well as provide a method to study older animals or others that do not demonstrate an adequate startle response. This is especially important in age-related studies of tinnitus, and for testing pharmacological or other treatments in animals that exhibit hearing loss in a longitudinal research. Our results provide further evidence that tinnitus results in a significant change in brainstem auditory function, including hyperactivity in the CN and IC. Many of the observed changes following SS-induced tinnitus were frequency specific, highlighting the tonotopic alterations in these structures. Temporal processing changes were also observed for all testing procedures, consistent with previous human and animal studies of tinnitus. Finally, our results should provide the impetus for further research that examines relationship between tinnitus and temporal processing deficits and whether they are related to the maladaptive plasticity that alters central gain following changes in peripheral input.

## Supporting Information

S1 Fig PPI to a 50 ms gap using a narrow band, 16 kHz gap carrier (shown in Fig. 1) compared to a broadband noise (BBN) gap carrier. We ran another experiment using the same protocol outlined for the multi-frequency gap-PPI, but with a BBN gap carrier to determine if gap detection would be impaired with a broadband signal. While gap detection was again reduced in response to the 16 kHz carrier, there was no effect following tinnitus induction when the BBN carrier was used.
(TIF)

S2 Fig ABR Threshold shift from baseline following SS-induced tinnitus (250 mg/kg) plotted as a function of tone burst frequency from 6–36 kHz. Threshold shift was fairly constant for frequencies between 6 and 24 kHz, with a difference of 3.4 dB between the highest and lowest change in threshold.
(TIF)

S3 Fig Correlation between the behavioral (gap-PPI) and electrophysiological (P2/P1 amplitude ratio) measures of tinnitus for the 16 kHz stimuli. The behavioral assay represents a shift from baseline for gap-PPI, where a positive shift (i.e. reduced gap-PPI) indicates tinnitus, and a negative shift indicates improved gap detection. The physiological assay represents the percent change in P2/P1 amplitude, where a shift from baseline in the negative direction (i.e. increased central to peripheral hyperactivity) also indicates tinnitus. In this case a value of 0 would indicate that the P2/P1 amplitude ratio was the same following tinnitus induction. Only the response to 16 kHz is shown, as it was the only stimulus where a statistically significant linear regression correlation was present.
(TIF)
